# Ti_3_C_2_T_x_-Modified PEDOT:PSS Hole-Transport Layer for Inverted Perovskite Solar Cells

**DOI:** 10.3390/molecules27217452

**Published:** 2022-11-02

**Authors:** Israt Ali, Muhammad Faraz Ud Din, Daniele T. Cuzzupè, Azhar Fakharuddin, Hitler Louis, Ghulam Nabi, Zhi-Gang Gu

**Affiliations:** 1 Fujian Institute of Research on the Structure of Matter, Chinese Academy of Sciences, Fuzhou 350002, China; 2 University of Chinese Academy of Sciences, Beijing 100049, China; 3 Institute of Physics, Slovak Academy of Sciences, 845 11 Bratislava, Slovakia; 4 Department of Physics, University of Konstanz, 78464 Konstanz, Germany; 5 Computational and Bio-Simulation Research Group, University of Calabar, Calabar 1115, Nigeria; 6 Energy Materials Lab (Physics), University of Gujrat, Gujrat 50700, Punjab, Pakistan

**Keywords:** perovskite solar cells, Ti_3_C_2_T_x_, PEDOT:PSS HTL, work function tuning

## Abstract

PEDOT:PSS is a commonly used hole-transport layer (HTL) in inverted perovskite solar cells (PSCs) due to its compatibility with low-temperature solution processing. However, it possesses lower conductivity than other conductive polymers and metal oxides, along with surface defects, limiting its photovoltaic performance. In this study, we introduced two-dimensional Ti_3_C_2_T_x_ (MXene) as an additive in the PEDOT:PSS HTL with varying doping concentrations (i.e., 0, 0.03, 0.05, and 0.1 wt.%) to tune the electrical conductivity of PEDOT:PSS and to modify the properties of the perovskite film atop it. We noted that the grain size of the CH_3_NH_3_PbI_3_ (MAPI_3_) perovskite layer grown over an optimal concentration of MXene (0.03 wt.%)-doped PEDOT:PSS increased from 250 nm to 400 nm, reducing charge recombination due to fewer grain boundaries. Ultraviolet photoelectron spectroscopy (UPS) revealed increased work function (WF) from 4.43 eV to 4.99 eV with 0.03 wt.% MXene doping, making the extraction of holes easier due to a more favorable energy level alignment with the perovskite. Quantum chemical investigations based on density functional theory (DFT) were conducted at the ωB97XD/6-311++G(d,p) level of theory to provide more insight into the stability, bonding nature, and optoelectronic properties of the PEDOT:PSS–MXene system. The theoretical investigations revealed that the doping of PEDOT:PSS with Ti_3_C_2_T_x_ could cause a significant effect on the electronic properties of the HTL, as experimentally demonstrated by an increase in the electrical conductivity. Finally, the inverted PSCs employing 0.03 wt.% MXene-doped PEDOT:PSS showed an average power conversion efficiency (PCE) of 15.1%, up from 12.5% for a reference PSC employing a pristine PEDOT:PSS HTL. The champion device with a 0.03 wt.% MXene–PEDOT:PSS HTL achieved 15.5% PCE.

## 1. Introduction

Organic–inorganic hybrid perovskite (e.g., CH_3_NH_3_PbI_3_)-based PSCs have attracted tremendous attention in the past few years [1]. Their exceptional power conversion efficiency (PCE) has driven the development of organic–inorganic halide perovskite solar cells [2]. Planar architectures of PSCs, which employ low-temperature-processable compact charge-transport layers, offer efficient, cost-effective, and facile processing [3] and have shown a certified PCE of 25.5% [4]. In general, the PCE of PSCs depends on the conductivity, work function, and energy level alignment of the charge-transport layers as well as the properties of the absorber layer, such as the chemical composition, bandgap, defects, and grain size of the perovskite absorber layer [5]. In terms of morphology, the larger grain size of MAPbI_3_ offers improved charge-carrier transport because of having fewer grain boundaries [6]. In inverted PSCs, the properties of the perovskite–HTL interface play a crucial role in determining the photovoltaic performance as well as the operational stability of the PSCs [7].

Various strategies have been employed to improve the interfaces in the PSCs as well as the properties of the perovskite bulk, including post-treatment, antisolvent engineering, incorporation of additives, and the use of hydrophilic charge-transport layers [8]. Extensive studies have focused on improving the electron-transport layer (ETL)’s properties and its interface with perovskite by utilizing different materials, including polymers, metal oxides, small molecules, and composite materials [9,10]. Recently, reports on optimizing the HTL–perovskite interface and the properties of HTLs have also attracted considerable interest in the field. It has recently been demonstrated that the hydrophilicity and wettability of the HTL can influence the grain size of the MAPbI_3_ layer, leading to an increase in conductivity and, thus, more efficient devices [11]. Modifying the HTL with polyphenylene sulfide has also been shown to increase the grain size of the perovskite layer and effectively improve the overall performance of the PSCs [12]. Poly(3,4-ethylenedioxythiophene): poly(styrenesulfonate) (PEDOT:PSS) is one of the most widely studied HTL materials for planar PSCs, owing to its tunable conductivity and good solution processibility [13]. However, it leads to limited open-circuit voltage and charge extraction due to a high surface defect density and a mismatch in energy levels with most common perovskite materials. Various strategies to improve the performance of PEDOT:PSS HTLs in PSCs include the use of bilayer structures, surface modification, doping with graphene oxide, etc. [14]. For instance, Niu et al. developed a graphene-oxide-doped PEDOT:PSS HTL that significantly improved hole extraction, resulting in a PCE of 20% [15]. A CuSCN-doped PEDOT:PSS HTL has also been shown to improve the morphology of the perovskite layer deposited on top and led to a higher work function [16].

MXenes emerged as a new class of 2D materials consisting of transition metal carbides/nitrides and carbonitrides obtained from ternary ceramic materials (MAX) by selectively etching A from them (M represents a transition element such as Ti, V, Nb, or Ta; A represents aluminum or silicon; and X represents carbon or nitrogen) [17]. MXenes, owing to their exceptional conductivity, high mobility, charge-carrier density, and excellent electronic properties, are utilized in inverted PSCs as HTLs and as dopants in the HTLs to improve the interfacial energy alignment, reduce surface defects, and improve the morphology of the perovskite deposited on top [18]. For instance, doping of PEDOT:PSS with Mo_1.33_C efficiently improved the charge-transport properties and increased the PCE by about 13% compared to its reference device [19]. Similarly, Wang et al. synthesized a Ti_3_C_2_-MXene-doped silane coupling agent vinyl Tris(2-methoxyethoxylsilane) (SCA)-based HTL [20]. The MXene-doped HTL improved the conductivity and hole extraction and led to an improved device.

In this work, we prepared Ti_3_C_2_T_x_-doped PEDOT:PSS as an HTL for inverted PSCs. The hydrophilicity of the Ti_3_C_2_T_x_-doped PEDOT:PSS HTL efficiently increased the grain size of the perovskite layer. Furthermore, an increase in the conductivity of the PEDOT:PSS-based HTL by doping with 0.03 wt.% Ti_3_C_2_T_x_ improved the charge extraction in the PSCs. We also thoroughly investigated the interaction of PEDOT:PSS with Ti_3_C_2_T_x_, along with its stability and optoelectronic properties, via density functional theory (DFT) calculations. The theoretical studies revealed that the doping of PEDOT:PSS with Ti_3_C_2_T_x_ could cause a significant effect on the electronic properties of the MXene-doped PEDOD:PSS. Moreover, the enhanced grain size of the perovskite layer on top also contributed to the PCE. Finally, the PSCs employing 0.03 wt.% Ti_3_C_2_T_x_-doped PEDOT:PSS showed a PCE of 15.1%—up from 12.5% for reference PSCs employing a pristine PEDOT:PSS HTL—while the champion device with 0.03 wt.% MXene-doped PEDOT:PSS achieved 15.5% PCE.

## 2. Results and Discussion

**Figure 1:**
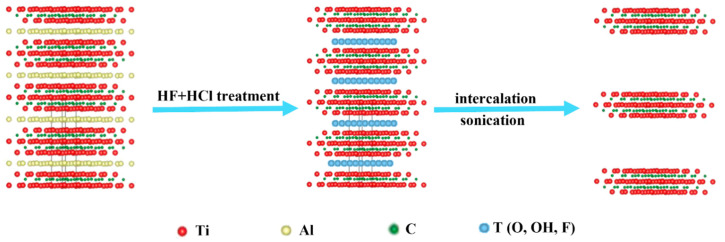
Schematics showing the synthesis of Ti_3_C_2_T_x_ nanosheets by a mixed-acid (LiF + HCl) method.

**Figure 2:**
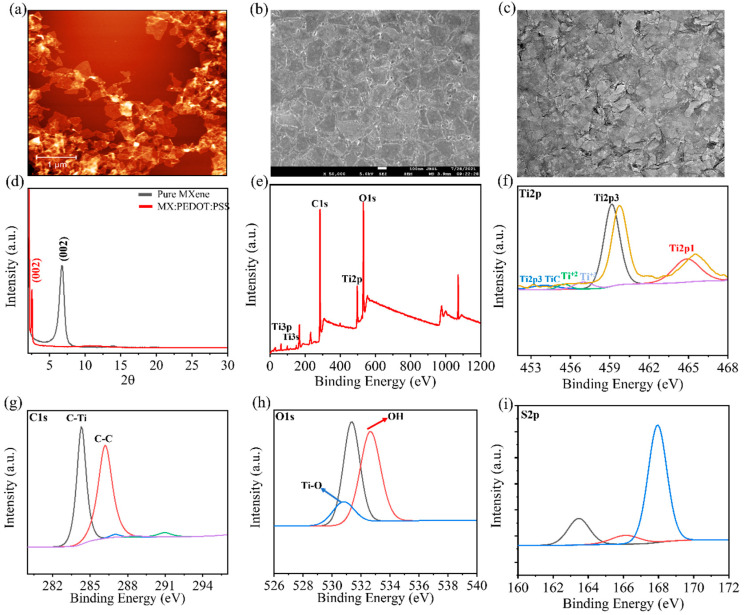
(**a**) AFM and (**b**) SEM images of well-distributed Ti_3_C_2_T_x_ nanosheets (scale bar: 100 nm), and (**c**) TEM image of Ti_3_C_2_T_x_ nanosheets (scale bar: 100 nm); (**d**) XRD patterns of pure Ti_3_C_2_T_x_ and Ti_3_C_2_T_x_-doped PEDOT:PSS; (**e**) XPS spectral survey of Ti_3_C_2_T_x_-doped PEDOT:PSS, and high-resolution XPS spectra of (**f**) Ti2p, (**g**) C1s, (**h**) O1s, and (**i**) S2p.

Two-dimensional Ti_3_C_2_T_x_ MXene nanosheets were synthesized from their MAX phase (Ti_3_AlC_2_) by etching Al using LiF + HCl (Figure 1). The multilayered Ti_3_C_2_T_x_ MXene sheets were bath-sonicated for 24 h to produce well-dispersed, single-layered, uniform nanosheets (Figure S1). Furthermore, AFM and SEM images confirmed the formation of a random distribution of MXene nanosheets after successful delamination and etching of Al layers (Figure 2a–c). The Ti_3_C_2_T_x_ nanosheets obtained after etching showed thicknesses of 30–90 nm and a size in the micrometer range (Figure S1). The presence of the main phase (002) in the XRD pattern indicated the efficient etching of the Al layer and an effective increase in the interlayer distance.

A strong peak at 2θ = 6.7° in the X-ray diffraction (XRD) pattern revealed the presence of a single nanosheet structure of Ti_3_C_2_T_x_ nanosheets, which is consistent with the available literature [21]. In contrast, this peak appeared in the MXene–PEDOT:PSS spectra with a slight shift because of the formation of a stable composite suspension, confirming that the MXene structure was maintained even with the addition of PEDOT:PSS (Figure 2d). The elemental distribution and surface chemical composition of MXene–PEDOT:PSS was analyzed by XPS spectra (Figure 2e). The spectra showed signals for elemental valences (i.e., C1s, O1s, S2p, and Ti2p) without the presence of any Al. No noticeable change was observed in the MXene-doped PEDOT:PSS, as expected for such a low doping concentration. In the high-resolution XPS spectra of Ti2p, peaks situated at 454.2, 455.4, 456.9, and 459.1 eV were assigned to Ti-C, Ti^+2^, Ti^+3^, and Ti-O, respectively (Figure 2f). Furthermore, analysis of the C1s element revealed two prominent peaks for C-Ti and C-C located at 284.2 and 286.2, respectively (Figure 2g). In the case of O1s, two peaks at 530.8 and 532.6 eV corresponded to Ti-O and C-Ti-(OH) bonding groups, respectively (Figure 2h). Finally, the high-resolution XPS spectra of S2p showed corresponding PSS and PEDOT peaks at binding energies of 167.9 and 163.4 eV, respectively (Figure 2i).

### 2.1. Computational Details

All computational calculations in this work were carried out using the Gaussian 16 and GaussView 6.0.16 suite of programs within a density functional theory (DFT) electronic structure framework. The ground-state optimization of the modeled structures, as presented in Figure S2, was conducted at the ωB97XD dispersion functional with the 6-311++G(d,p) basic set. By invoking Gaussian 16, natural bond orbitals (NBOs), which represent the stabilization of inter- and intramolecular charge transfer between molecules and their respective occupancies, were computed using the inbuilt Gaussian 3.1 methods available in Gaussian 16 software. To further understand the nature of interactions within the polymer cluster, the quantum theory of atoms in molecules (QTAIM) was computed using multifunctional wave-function analysis developed by Tian Lu’s research group and downloaded from an official webpage (http://sobereva.com/Multiwfn). The UV excitation energy calculation was simulated within the Franck–Condon excitation (S_0_ → S_1_) by utilizing the TD-SCF/CPCM/ωB97XD/6-311++G(d,p) method in water as the solvation medium.

### 2.2. HOMO–LUMO Analysis

To obtain deeper understanding of the reactivity and stability of the studied compounds and their levels of interaction, frontier molecular orbital (FMO) analyses were employed using the log file from the geometric optimization of the respective compounds [22]. The highest occupied molecular orbital (HOMO) and lowest unoccupied molecular orbital are the central orbitals that constitute the frontier molecular orbital. When linking the FMO and Koopmans’ approximation, it is essential to note that the HOMO energy level plays a role as an electron donor. In contrast, the LUMO energy level deals with electron acceptance, which can be referred to as electron affinity [23]. The difference in energy between the highest occupied molecular orbital (HOMO) and the lowest unoccupied molecular orbital (LUMO) is generally regarded as an energy gap expressed in electron volts (Eg/eV) or bandgap. The latter is a critical parameter that gives complete information about compounds’ reactivity and stability; thus, a small energy gap depicts high reactivity and less kinetic stability, while a large energy gap connotes high stability and less reactivity of such compounds. As presented in Table 1, we computationally studied three different phases, and the results obtained—as shown in Table 1—have a reactivity trend of interaction > polymer > cluster, with energy gap values of 1.828 eV, 2.171 eV, and 6.141 eV, respectively; from these results it is clear that the interaction of the cluster and the polymer was more reactive compared to the other phases. The MXene cluster optimization was very stable due to the atoms involved in the cluster formation; the electronegativity of the oxygen atoms in the polymer structure makes it more reactive than the cluster optimization. Comparatively, the interaction energy of 1.838 eV was more reactive due to the charge transfer and the bonding nature of the cluster with the polymer structure [23]. This cluster kinetic stability was also observed in the second-order stabilization energy. A pictorial representation of the HOMO and LUMO is presented in Figure S3 to help understand the distribution of the electrons within the molecules. From this representation, it is worth noting that the electron distribution in the interaction between the cluster and polymer in the HOMO was highly concentrated on the cluster; this was observed as the cluster was overshaded, leaving the polymer unshaded. This observation could be explained on the basis of the cluster being electron-rich compared to the polymer, whereas the LUMO was distributed across the interacting species.

### 2.3. Natural Bond Orbital Analysis

Natural bond orbital (NBO) analysis is an important parameter that is used to computationally ascertain the type of donor (filled) and acceptor (unfilled) orbital interactions persisting within a molecule. NBO analysis of the studied compounds was executed using the density functional theory method at the DFT/wB97XD/6-311++G(d,p) level of theory to validate the intra- and intermolecular hyperconjugation, electron delocalization, and charge transfer existing within the studied compounds, and to understand the occupancies of the studied interaction to further validate the intermolecular charge transfer. The strength of the interaction between the donor (filled) and acceptor (unfilled) was represented using second-order perturbation energy. The second-order perturbation energy was evaluated using Equation (1):

(1) $$E^{\left(2\right)}=\Delta E_{i,j}=-q_{i}\frac{F^{2}\left(i,j\right)}{\varepsilon_{i}-\varepsilon_{j}}$$

where $q_{i}$ is the donor occupancy, $\varepsilon_{i}$ and $\varepsilon_{j}$ signify the diagonal elements, and $F_{\left(i,j\right)}$ is the Fock matrix. Table 2 presents the inter/intramolecular charge transfer, the highest occupancies of the charges, and the second-order stabilization energy of the respective compounds. Considering the stabilization energy, the highest five energy levels of the compounds were highly studied. From the results, the strength of interactions of the studied compounds, which are expressed as second-order perturbation energy $E^{\left(2\right)}$ were found to follow the trend cluster > interaction > polymer, with stabilization energy of 542.56 kcal/mol, 299.45 kcal/mol, and 40.08 kcal/mol, respectively, further confirming the stability of the cluster compared to the other phases. The maximum interactions with the highest $E^{\left(2\right)}$ for the cluster were LpC_2_–p* Ti_23_ (542.56 kcal/mol) and σ^*^C_1_–Ti_19_ → σC_1_–Ti_23_ (469.80 kcal/mol), while for the polymer the interactions with the highest stabilization energy were πC_28_–C_31_ → π*C_26_–C_27_ (40.08 kcal/mol) and πC_29_–C_33_ → π*C_28_–C_31_ (38.17 kcal/mol). Similarly, the strongest interaction resulting in the highest stabilization energy in the interaction was observed for π*C_44_–Ti_66_ → πC_45_–Ti_66_ (299.45 kcal/mol) and Lp*Ti_66_ → Lp*Ti_65_ (239.05 kcal/mol). These higher interactions were observed to have charge occupancies of 1.08977 and 1.77480 for the cluster, respectively, and 1.43478 and 1.66383 for the polymer, respectively. Similarly, the occupancies for the cluster and polymer interaction were 1.61583 and 1.43721, respectively. The NBO analysis showed that the cluster had the highest occupancy value, which was correlated with the higher stability of the cluster and the charge transfer, and was thereby responsible for the stabilization energy. The highest stabilization energy in this study was observed from the lone pair of electrons. Hydrogen bonds were expected to arise through charge transfer from the lone pair (LP) of proton acceptors to the anti-bonding of the proton donor. Thus, the amount of charge transfer and the energy of interaction were more than has been observed in these studies. Conclusively, the larger the second-order perturbation energy value of $E^{\left(2\right)}$, the greater the extent of charge transfer from the Lewis-type donor to non-Lewis-type acceptors; as such, based on our findings, the cluster had the highest $E^{\left(2\right)}$ value (542.56 kcal/mol).

### 2.4. Quantum Theory of Atoms in Molecules (QTAIM)

To further investigate the bond formation and the nature of the interaction between the studied polymer and the cluster, topological properties such as the density of all electrons (e(r)), Laplacians of electron densities ∇e(r), Lagrangian kinetic energy (G(r)), potential energy density (V(r)), Hamiltonian kinetic energy, and electron localization density at the bond critical points (BCPs) were calculated using Bader’s theory of atoms in molecules. Based on the general knowledge, e(r) > 0 denotes the weak covalent interactions (strong electrostatic bonds), while ρ0 represents the medium-strength or partially covalent bonds. In Table 3, the e(r) values for all interactions are positive and indicate a robust electrostatic type of interaction between the cluster and the polymer. As shown in Figure S4, four different interactions were observed between the polymer and the cluster. The most significant interaction observed here was intermolecular bonding. At the same time, intramolecular bonding was also observed in the cluster. The electron localization function (ELF) is essential in evaluating the covalent bond interaction. When the ELF value is between 0.5 and 1, it suggests regions with bonding and nonbonding localized electrons. When the ELF value is less than 0.5, the electron is delocalized. ELF values for interactions between cluster and polymer results are higher than 0.5, indicating bonding and nonbonding localized electrons. ELF values for other interactions within only the polymer are delocalized and partially covalent. The balance between G(r) and V(r) shows the nature of the interaction; the most significant and the highest topological values calculated in this study are presented in Table 3. Based on this study’s findings, the density of electron values for the interaction at BCPs 92, 104, and 127 was observed to be 0.1425e, 0.9501e, and 0.1063e, respectively. Furthermore, this electron density was observed to have the range 0.0362e–0.844e. For the polymer, the most interaction observed was intramolecular bonding interaction, as shown in Figure S3; the most interesting bond critical points observed were 49, 63, and 73, with electron densities of 0.1828e, 0.1840e, and 0.3704e, respectively. This electron density was observed to have a range of −0.0012 −0.1864e. It was anticipated that strong bonds would be related to higher electron density, as seen in the interaction between H16 and C23, which reflects the strong bonding between the cluster and the polymer. The positive values of ELF indicate that the cluster’s interaction with the polymer is electrostatic. This nature of the interaction was further investigated with the Laplacian of electron density.

### 2.5. UV Excitation Analysis

The excitation type, energy, wavelength, oscillator strength, and significant orbital contributions of the titled molecules were investigated using water as the solvent, and the results are displayed in Table 4. The wavelength in the studied compounds was observed to follow the order interaction > cluster > polymer, with (λ_max_ nm) values of 511.10 nm, 490.17 nm, and 240.14 nm, respectively. The studied compound had vertical excitation energies of 5.1629 eV, 2.5294 eV, and 2.258 eV, which were associated with oscillator strengths of 0.1970, 0.0000, and 0.0006 for the polymer, interaction, and cluster, respectively. The prominent intense absorption (34.9% orbital contributions) arose because of electronic transitions from the orbital index of 165 → 169 for the cluster. Moreover, the most significant absorption observed from the polymer (6.885% orbital contributions) was from the orbital transition of 97 → 100. Interestingly, it is worth noting that the highest contribution observed in this study was from the interaction of the Ti_3_C_2_T_x_-cluster with the polymer, which presented 265 → 266 (61.60% orbital contribution) to be the maximum absorption site. This higher contribution was observed to be correlated with the higher wavelength of this interaction, which is consistent with the energies of the frontier molecular orbitals [24].

**Figure 3:**
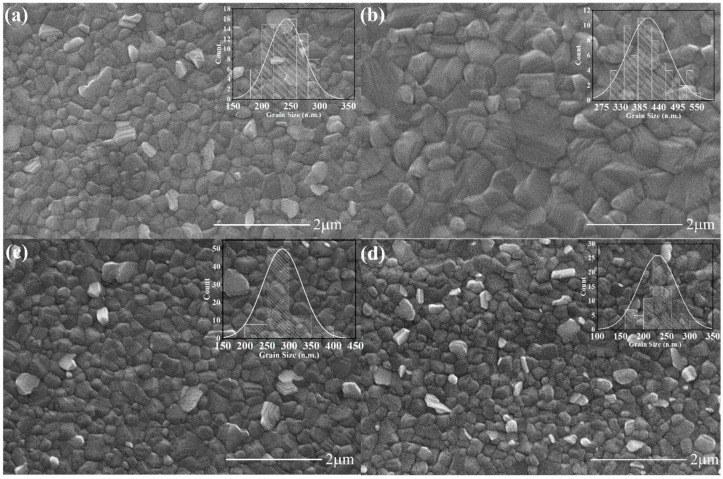
SEM images of perovskite films deposited over PEDOT:PSS with Ti_3_C_2_T_x_ doping concentrations of (**a**) 0 wt.%, (**b**) 0.03 wt.%, (**c**) 0.05 wt.%, and (**d**) 0.1 wt.% (the insets show crystal size measurements of perovskite using ImageJ software).

SEM images show the quality of the various perovskite films deposited on top of the various HTLs. We observed a uniform morphology for all of the perovskite films without any pinholes (Figure 3a–d). A pronounced increase in the size of the perovskite crystals from 250 nm to 400 nm was observed for the 0.03 wt.% Ti_3_C_2_T_x_-doped PEDOT:PSS HTL. However, upon depositing the perovskite layer over 0.05 wt.% and 0.1 wt.% Ti_3_C_2_T_x_-doped PEDOT:PSS HTLs, the crystal size measured using ImageJ was 290 nm and 250 nm, respectively (Figure 3c,d). A visible decrease in the grain size of perovskite was caused by the loss of hydrophilicity of the HTL. Generally, an increase in perovskite crystal size was observed using an appropriate concentration of MXene as a dopant with PEDOT:PSS, as compared to the undoped PEDOT:PSS. The enhanced grain size can be attributed to the growth of perovskite over the randomly distributed MXene flakes, which can slow the crystallization rate of the perovskite. At a specific weight percentage of MXene—i.e., 0.03 wt.% in the HTL—the larger grain size of perovskite crystals was observed because of the hydrophilic nature of the HTL, which can lead to an improved PCE [25]. Surface roughness is another important parameter that determines the quality of the perovskite films deposited atop the HTL. The AFM data (Figure S5) suggest that the surface roughness drops upon the inclusion of Ti_3_C_2_T_x_ in the PEDOT:PSS. The root-mean-square roughness dropped from 1.66 nm for the pristine PEDOT:PSS film to 0.89 and 0.84 nm for 0.03 and 0.05 wt.% Ti_3_C_2_T_x_ doping, respectively. A further increase in the Ti_3_C_2_T_x_ doping led to another increase in the surface roughness (rms value of 1.06 nm). Furthermore, the XRD analysis showed similar crystallinity of the perovskite over the MXene-doped HTLs (Figure S9 and Table S2).

**Figure 4:**
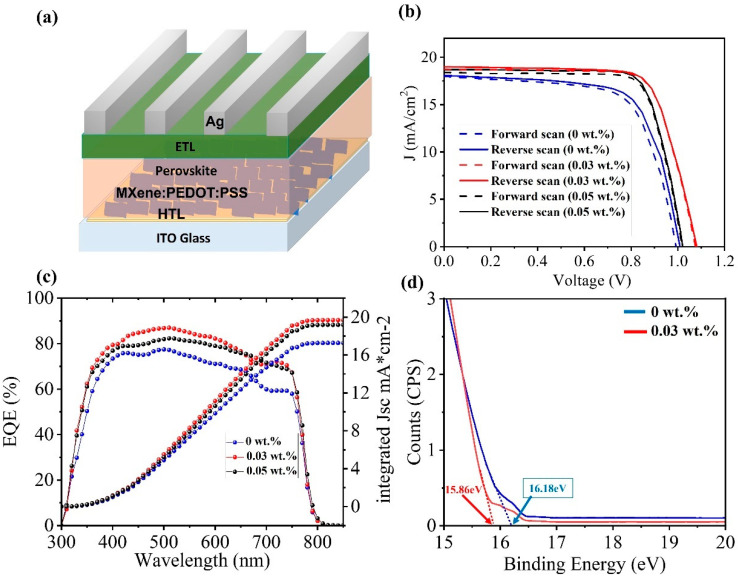
(**a**) Device structure of the inverted PSCs employing PEDOT:PSS (pristine or MXene-doped) as an HTL and PC_61_BM as an ETL. (**b**) J–V curves of PEDOT:PSS, 0.03 wt.% Ti_3_C_2_T_x_-doped PEDOT:PSS, and 0.05 wt.% Ti_3_C_2_T_x_-doped PEDOT:PSS devices under different scan directions. (**c**) Incident photon conversion efficiency (IPCE) spectra and integrated J_sc_ for different doping concentrations of Ti_3_C_2_T_x_. (**d**) UPS spectra for pure PEDOT:PSS and Ti_3_C_2_T_x_-doped PEDOT:PSS.

We investigated the effects of Ti_3_C_2_T_x_-doped PEDOT:PSS HTLs in PSCs (device configuration ITO/MXene:PEDOT:PSS/MAPbI_3_/PC_61_BM/BCP/Ag) (Figure 4a). The SEM cross-sectional image of the device is shown in Figure S8. The current density–voltage (*J–V*) curves under 1-sun illumination conditions (i.e., light intensity of 100 mW/cm^2^, AM 1.5G) are shown in Figure 4b. The PSCs with PEDOT:PSS as the HTL showed lower J_sc_ and V_oc_ than those employing Ti_3_C_2_T_x_-doped PEDOT:PSS HTLs. A possible reason for the lower PCE in the case of a pure PEDOT:PSS HTL is its inferior hydrophilicity and poor hole-extraction efficiency. The highest PCE was recorded for the 0.03 wt.% Ti_3_C_2_T_x_-doped PEDOT:PSS HTL. A further increase in the concentration of Ti_3_C_2_T_x_ to 0.05 wt.% lowered the J_sc_ and V_oc_ compared to the 0.03 wt.% doping concentration (Table 5). This decrease in PCE when increasing the concentration of Ti_3_C_2_T_x_ in PEDOT:PSS was attributed to an increase in surface roughness and the non-homogenous HTL layer (Figure S5). These data show that the 0.03 wt.% Ti_3_C_2_T_x_ doping led to a substantial improvement in the overall performance of the PSC.

The increased J_sc_ value for the 0.03 wt.% Ti_3_C_2_T_x_-doped HTL was supported by the IPCE measurements (Figure 4c). The IPCE spectra for different doping concentrations of Ti_3_C_2_T_x_ revealed that the highest charge collection was for the optimized (0.03 wt.%) concentration of Ti_3_C_2_T_x_ in the HTL. The increased IPCE value in the 450–700 nm region indicates the higher light absorption and extraction ability of the HTL because of the appropriate doping of Ti_3_C_2_T_x_. To understand the reasons behind the improved charge extraction, we measured the UPS spectra for pristine PEDOT:PSS and 0.03 wt.% Ti_3_C_2_T_x_-doped PEDOT:PSS. The Ti_3_C_2_T_x_-doped PEDOT:PSS film exhibited a higher work function (WF) of 4.99 eV as compared to the pristine PEDOT:PSS film (4.43 eV), which is advantageous for extracting holes from the perovskite layer to the HTL in PSCs (Figure 4d). The increased work function leads to a better band alignment with the perovskite, resulting in better charge extraction.

**Figure 5:**
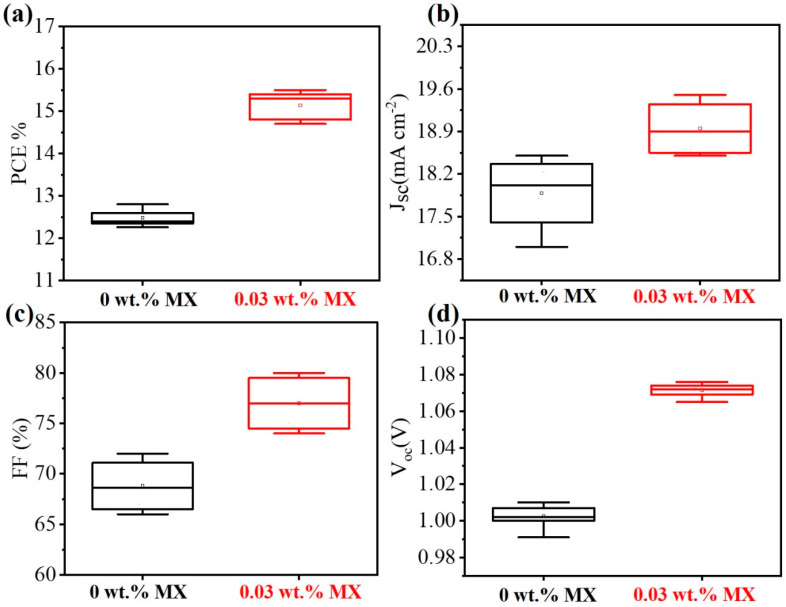
Statistical parameters of PSCs with pure PEDOT:PSS and 0.03 wt.% Ti_3_C_2_T_x_-doped PEDOT:PSS (reverse scan): (**a**) power conversion efficiency (PCE); (**b**) short-circuit current density (J_sc_); (**c**) fill factor (FF); (**d**) open-circuit voltage (V_oc_). Approximately 10 devices for each parameter were statistically analyzed.

We also showed the reproducibility of PSCs employing pristine and 0.03 wt.% Ti_3_C_2_T_x_-doped HTLs in 10 devices, and their photovoltaic performance is summarized in Figure 5a–d. The average PCE, J_sc_, FF, and V_oc_ were enhanced in the 0.03 wt.% Ti_3_C_2_T_x_-doped HTL. These increases can be attributed to the surface hydrophilicity and reduced surface roughness of Ti_3_C_2_T_x_-doped HTL, which significantly impacted the increase in grain size of MAPbI_3_ crystals, as well as improving the conductivity and increasing the work function.

**Figure 6:**
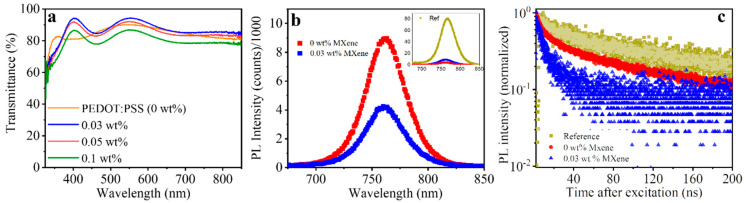
(**a**) Transmission spectra of PEDOT:PSS (0 wt.% MXene) compared with various doping concentrations of MXenes in PEDOT:PSS. (**b**) Steady-state photoluminescence spectra of perovskite films deposited on PEDOT:PSS (0 wt.% MXene) and 0.03 wt.% MXene-doped PEDOT:PSS; the inset shows the photoluminescence of the same compared with that of a perovskite film deposited on top of a non-quenching glass substrate. (**c**) Time-resolved photoluminescence decay transients of the various perovskite films. The perovskite films were excited from the glass side (HTL–perovskite interface) with a pulsed laser (λ_exc_ 405 nm).

In order to measure the optical properties of MXene-doped PEDOT:PSS thin films, the transmission of the films was recorded (Figure 6a). The 0.03 and 0.05 wt.% MXene-doped films showed slightly higher transmittance, which was attributed to interference effects. Higher concentrations of MXene doping led to a drop in the transmittance. This trend was further visible upon an increase in the MXene doping to 0.2 and 0.5 wt.%, with both films showing a further decrease in transmittance (Figure S6). The perovskite films deposited atop these various HTLs showed similar transmittance (Figure S7), suggesting identical optical properties of all of these perovskite films. This, together with the similar surface morphology of the perovskite films (Figure S5), suggests that the Ti_3_C_2_T_x_ doping in the PEDOT:PSS did not have a significant effect on the surface roughness and optical properties of the perovskite, and that any change in the PV performance due to these two factors can be negligible.

To probe the influence of the Ti_3_C_2_T_x_ doping on the recombination and charge extraction from the perovskite layer, steady-state photoluminescence (PL) and time-resolved PL of the perovskite films deposited on top of the pristine PEDOT:PSS and that with the optimal concentration of Ti_3_C_2_T_x_ doping (0.03%) were carried out (Figure 6b,c). The perovskite films deposited on both of these HTLs showed nearly an order of magnitude drop in the PL intensity (inset in Figure 6b), which can be attributed to quenching of charge carriers. Notably, the 0.03 wt.% MXene doping showed a reduced PL intensity, which is an indication of a better charge extraction due to a more favorable energy level alignment. This is further reflected in the time-resolved PL (TRPL) measurements. The TRPL transients of the 0.03% Ti_3_C_2_T_x_-doped sample show a pronounced drop in the PL during the first few ns, which can be attributed to charge transfer from the perovskite to the HTL. This initial drop was more pronounced for the MXene-doped sample than its pristine PEDOT:PSS counterpart, indicating a more efficient charge transfer from the former.

**Figure 7:**
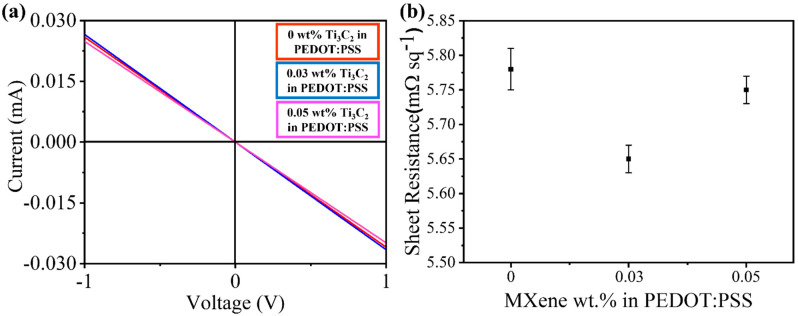
(**a**) I-V measurement, (**b**) Sheet resistance of 0 wt.%, 0.03 wt.%, and 0.05 wt.% Ti3C2Tx-doped PEDOT:PSS.

Conductivity is another critical parameter for a charge-transport layer to work efficiently in solar cells, and appropriate doping should lead to enhanced conductivity. In our case, PEDOT:PSS HTLs were doped with different concentrations of Ti_3_C_2_ MXene, and it can be seen that the conductivity value was only increased for the 0.03 wt.% doping of Ti_3_C_2_. In contrast, the conductivity of the overall HTL dropped for lower or higher Ti_3_C_2_ doping concentrations (Figure 7a). This is because conductivity shows a trade-off between charge-carrier density and charge mobility (σ=qμn, where q is the elementary charge, μ is the carrier mobility, and *n* is the charge-carrier density). Ideally, an HTL should have a high carrier mobility and a high hole concentration, which was likely the case in the 0.03 wt.% Ti_3_C_2-_doped HTL. Similarly, the resistance measured for the 0.03 wt.% doping concentration of Ti_3_C_2_ was lower than that measured for the pristine and 0.05% doped HTL (Figure 7b). These measurements suggest that the increased conductivity of the 0.03% doped HTL is another crucial factor contributing to the overall improved performance of the PSCs.

## 3. Experimental

### 3.1. Materials and Methods

PEDOT:PSS (99.9%), PC_61_BM (99.9%), and bathocuproine (99.9%) were purchased from Ossila. Chlorobenzene (99.8%), isopropyl alcohol (99%), dimethylformamide (99%) and dimethyl sulfoxide (99.9%) were purchased from Sigma-Aldrich. MAI (99.5%) was purchased from Deysol Sweden, while PbI_2_ (99.9%) was purchased from TCI chemicals.

Characterizations: UV–Vis–NIR absorption spectra were measured on a P7 double-beam UV–visible spectrophotometer (Shanghai, China). Transmission electron microscopy (TEM) images were acquired using a JEM-1400 Flash instrument operated at 80 kV (Tokyo, Japan), whereas the morphology of various materials in this work was investigated using a Hitachi S-4800 field-emission scanning electron microscope (SEM) and a Veeco Dimension 3100 V atomic force microscope. X-ray diffraction (XRD) patterns were obtained using a Bruker D8 X-ray diffractometer. X-ray photoelectron spectroscopy (XPS) data were obtained using an AXIS ULTRA DLD.

Atomic force microscopy (AFM) measurements of the various HTLs and perovskite films deposited atop them were performed using a Park Systems NX10 in air in a true non-contact mode. Photoluminescence measurements were performed using a PicoQuant setup using a pulsed laser with an excitation wavelength of 405 nm.

### 3.2. Synthesis of Ti_3_C_2_T_x_ MXene and Doping of PEDOT:PSS

Single-layer micro-sized Ti_3_C_2_T_x_ MXene sheets were synthesized using mixed acids (HCl + LiF) to etch aluminum (Al) from Ti_3_AlC_2_. Cutting of as-prepared larger Ti_3_C_2_T_x_ sheets into smaller ones was performed using bath sonication. The bath sonication (Sonorex RK 510 H, 35 kHz, Bandelin) was applied for 24 h in an ice bath. The nanosheets were centrifuged (Model 3–30K, Sigma centrifuge) at 8000 rpm for 40 min at 20 °C. The residue was collected and vacuum-dried overnight. Next, the thoroughly dried nanosheets were dissolved in 5 mL of water. The nanosheets were characterized by atomic force microscopy (AFM) and scanning electron microscopy (SEM). Lastly, different amounts of Ti_3_C_2_ MXene nanosheets were used to dope specific amounts of PEDOT:PSS and stirred for three hours at room temperature.

### 3.3. Device Fabrication

The inverted PSCs were fabricated using pre-patterned ~7 Ω ITO-coated glass. Initially, the ITO substrates were cleaned with Hellmanex solution, acetone, and isopropanol for 20 min each, followed by a UV–ozone treatment for 30 min. The PEDOT:PSS solution was spin-coated over the ITO substrate at 4000 rpm for 30 s and annealed for 35 min at 120 °C. Afterward, the substrates were immediately taken to the glovebox to deposit the perovskite layer.

Next, 1.2 M MAPI_3_ was prepared and stirred overnight at room temperature. The spin-coating of MAPI_3_ involved two steps: First, 60 μL of MAPI_3_ solution was spin-coated over the ITO substrate for 10 s at 1000 rpm, followed by 5000 rpm for another 45 s. After 15 s into the second step, 150 μL of chlorobenzene was dropped onto the spinning substrate to enable perovskite crystallization. Afterward, the MAPI_3_ was annealed at 100 °C for 20 min. The substrates were cooled down before spin-coating [6,6]-phenyl-C61-butyric acid methyl ester (PC_61_BM) ETL. 

Then, 50 μL of PC_61_BM was spun over the perovskite layer at 1000 rpm for 30 s, followed by deposition of the BCP layer (60 μL at 5000 rpm). Finally, 100 nm Ag was evaporated to complete the device.

## 4. Conclusions

In summary, the effects of Ti_3_C_2_T_x_ doping in PEDOT:PSS HTLs were studied for inverted perovskite solar cells. The 0.03 wt.% MXene doping not only showed an enhancement in the conductivity of the PEDOT:PSS compared to the undoped PEDOT:PSS, but also enhanced the grain size of MAPbI_3_ from 250 nm to 400 nm. Increasing the grain size is a useful strategy to decrease grain boundaries, which reduces the charge recombination at these boundaries. The theoretical investigations revealed that the doping of PEDOT:PSS with Ti_3_C_2_T_x_ could cause a significant effect on the electronic properties of the final product. Experimentally, it was proven that the combination of Ti_3_C_2_T_x_ and PEDOT:PSS is favorable for PSCs, resulting in an effective increase in PCE (15.5%) for the champion device. The synergistic effect of increased conductivity, work function, and MAPI_3_ crystals’ size resulted in an average enhancement of PCE from 12.5% to 15.1%.

## Figures and Tables

**Table 1:** HOMO–LUMO energy and energy gap of the studied compound according to DFT at wB97XD/6-311++G(d,p).

Phases	HOMO/eV	LUMO/eV	Energy Gap (e/V)
Cluster	−6.474	−0.333	6.141
Polymer	−4.951	−2.780	2.171
Interaction	−3.679	−1.841	1.838

**Table 2:** Second-order perturbation analysis and occupancies of the Fock matrix for the studied compounds based on DFT at wB97XD/6-311++G(d,p).

Phases	Donor (ἰ)	Acceptor (j)	$E^{\left(2\right)}$ (kcal/mol)	Occupancies	E(j)–E(ἰ) (a.u)	F(ἰ,j) (a.u)
**Cluster**	LpC_2_	Lp*Ti_23_	542.56	1.08977	0.08	0.209
	σ^*^C_1_–Ti_19_	σC_1_–Ti_23_	469.80	1.77480	0.06	0.235
	σ^*^C_1_–Ti_19_	Lp*Ti_19_	359.85	1.43478	0.04	0.194
	πC_1_–Ti_19_	πC_1_–Ti_23_	131.97	1.78948	0.05	0.102
	LpTi_23_	π*C_1_–Ti_23_	126.01	0.77931	0.05	0.086
**Polymer**	πC_28_–C_31_	π*C_26_–C_27_	40.08	1.67461	0.38	0.112
	πC_29_–C_33_	π*C_28_–C_31_	38.17	1.66383	0.38	0.109
	πC_26_–C_27_	π*C_29_–C_33_	36.21	1.64736	0.40	0.105
	πC_29_–C_33_	π*C_26_–C_27_	32.49	1.67461	0.38	0.099
	πC_26_–C_27_	π*C_28_–C_31_	30.44	1.66383	0.40	0.098
**Interaction**	π*c_44_–Ti_66_	πC_45_–Ti_66_	299.45	1.61583	0.01	0.073
	Lp*Ti_66_	Lp*Ti_65_	239.05	1.43721	0.01	0.121
	Lp*Ti_56_	Lp*Ti_57_	196.02	1.64411	0.01	0.120
	Lp*Ti_59_	Lp*Ti_56_	174.42	1.67911	0.01	0.106
	σ*C_46_–Ti_65_	Lp*Ti_65_	127.39	1.69166	0.12	0.259

**Table 3:** The properties of the topology analysis.

Compound	Bond	BCP	e (r)	∇ e(r)	G(r)	K(r)	V(r)	ELF
**Interaction**	H_12_–C_52_	92	0.1425	−0.1021	0.3056	0.2859	−0.3165	0.9926
	H_16_–C_48_	104	0.9501	0.2824	0.5721	−0.1338	−0.4383	0.4360
	O_7_–C_23_	127	0.2819	0.1063	0.1992	−0.6651	−0.1327	0.6469
**Polymer**	O_37_–H_10_	49	0.1828	0.6892	0.1456	−0.2668	−0.1189	0.5876
	C_31_–H_11_	63	0.1840	−0.3969	0.3772	0.1370	−0.1747	0.9535
	C_28_–O_7_	73	0.3704	0.1352	0.2712	−0.6685	−0.2044	0.8674

**Table 4:** Absorption energies, wavelengths (nm), and major contributions with oscillator strength for the studied molecules in the aqueous phase, calculated at TD-SCF/CPCM/wB97XD/6-311++G(d,p).

Compound	Excited State	Energy (eV)	Wavelength (nm)	Oscillator Strength (f)	Orbital Contribution (%)
**Cluster**	S_0_ → S_1_	2.5294	490.17	0.0000	165 → 169 = (34.9)
					165 → 17 = (12.581)
					166 → 170 = (39.16)
**Polymer**	S_0_ → S_1_	5.1629	240.14	0.1970	97 → 98 = (4.732)
					97 → 100 = (6.885)
					97 → 103 = (2.253)
**Interaction**	S_0_ → S_1_	2.4258	511.10	0.0006	264 → 267 = (3.773)
					265 → 266 = (61.60)
					265 → 268 = (5.518)

**Table 5:** Photovoltaic performance parameters of PSCs employing pristine PEDOT:PSS and different doping amounts of Ti_3_C_2_T_x_ -doped PEDOT:PSS as HTLs.

Dopant Concentration	V_oC_ (V)	J_sc_ (mA/cm^2^)	FF (%)	PCE (%) Reverse Scan	PCE (%) Forward Scan
0 wt.%	1.00 ± 0.01	18.01 ± 01	68.6 ± 04	12.5 ± 0.3	12.0 ± 0.2
0.03 wt.%	1.07 ± 0.01	19.02 ± 0.5	76.5 ± 03	15.1 ± 0.4	15.0 ± 0.3
0.05 wt.%	1.02 ± 0.01	18.70 ± 0.5	76.3 ± 04	14.5 ± 0.4	14.3 ± 0.4

## Data Availability

Not applicable.

## Supplementary Materials

The following supporting information can be downloaded at: https://www.mdpi.com/article/10.3390/molecules27217452/s1, Figure S1: AFM image of 24 h sonicated Ti_3_C_2_ nanosheets. Figure S2: Representation of the optimized structure of the cluster, polymer, and the cluster-polymer interaction. White, grey, red, and yellow represent titanium, carbon, oxygen, and sulfur atoms respectively. Figure S3: Pictorial representation of HOMO-LUMO energies and densities. Figure S4: Pictorial representation of the Optimized structure and the QTAIM. Figure S5: (top panel) AFM images of PEDOT:PSS films doped with various concentrations of MXenes. (bottom panel) AFM images of perovskite films deposited on top of the same HTLs, respectively. Figure S6: Transmittance spectrum of 0.2 wt.% doped MXene in PEDOT:PSS and a pristine PDOT:PSS layer on ITO. Figure S7: Transmission spectra of perovskite films on top of MXene-doped HTLs on ITO. Figure S8: Cross-sectional SEM image of the device. Figure S9: Comparison of the (110) and (220) XRD diffraction peaks. Table S1: Surface roughness of various HTL and perovskite films on HTLs. Table S2: XRD peak analysis.

## Sample Availability

Samples of the Ti_3_C_2_T_x_, PEDOT: PSS HTL are available from the authors.
