# Supplementary material for: Ti3C2Tx-Modified PEDOT:PSS Hole-Transport Layer for Inverted Perovskite Solar Cells
Source: Molecules. 2022 Nov 2;27(21):7452. doi: 10.3390/molecules27217452 (PMC9655112; doi:10.3390/molecules27217452)
Supplement: Supplementary file 1 [file molecules-27-07452-s001.zip › molecules-1961833-supplementary.pdf]

Supporting information

$Ti_3C_2T_x$ Modified PEDOT:PSS Hole Transport Layer for Inverted Perovskite Solar Cells

Israt Ali [1,2], Muhammad Faraz Ud Din [3], Daniele T. Cuzzupè [4], Azhar Fakharuddin [4], Louis Hitler [5], Ghulam Nabi [6], and Zhi-Gang Gu [1,2]

[1] Fujian Institute of Research on the Structure of Matter, Chinese Academy of Sciences, 350002 Fuzhou, China
[2] University of Chinese Academy of Sciences, Beijing 100049, China
[3] Institute of Physics, Slovak Academy of Sciences, 845 11, Bratislava, Slovak Republic
[4] Department of Physics, University of Konstanz, 78464 Konstanz, Germany
[5] Computational and Bio-Simulation Research Group, University of Calabar, Calabar, Nigeria
[6] Energy Materials Lab (Physics), University of Gujrat, Gujrat 50700 Punjab, Pakistan.

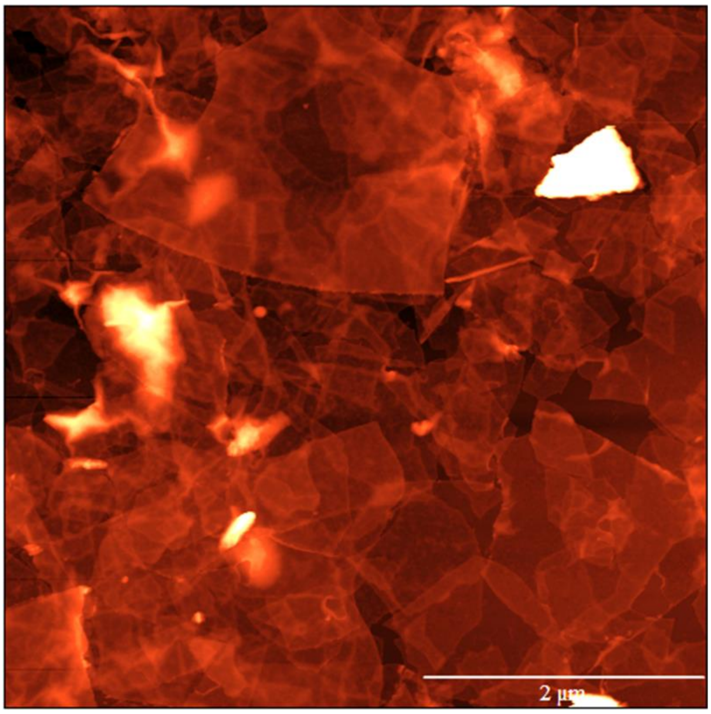

Figure S1. AFM image of 24 hours sonicated Ti_3C_2 nanosheets.

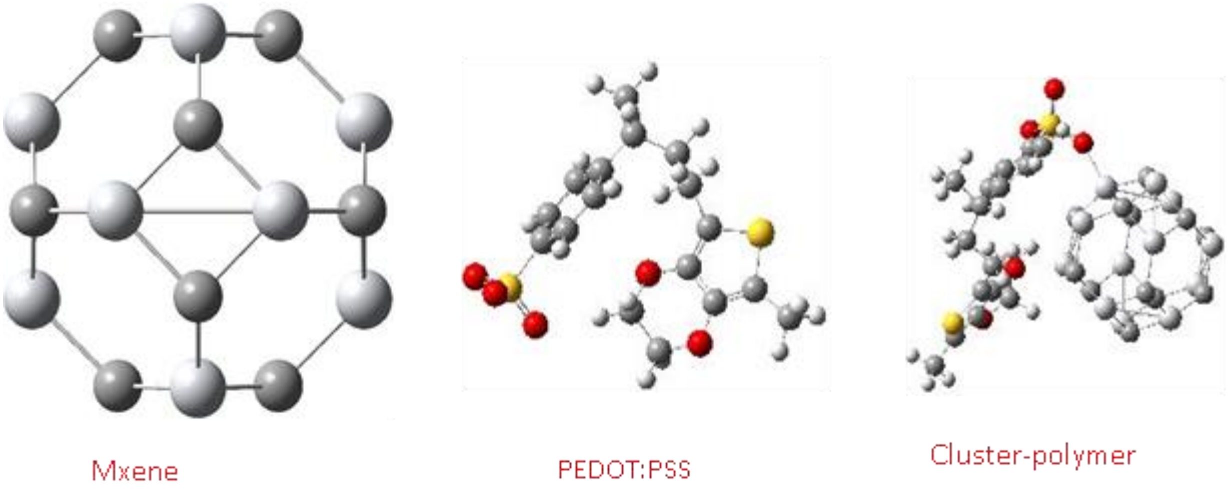

Figure S2. Representation of the optimized structure of the cluster, polymer, and the cluster-polymer interaction. White, grey, red, and yellow represent titanium, carbon, oxygen, and sulfur atoms respectively.

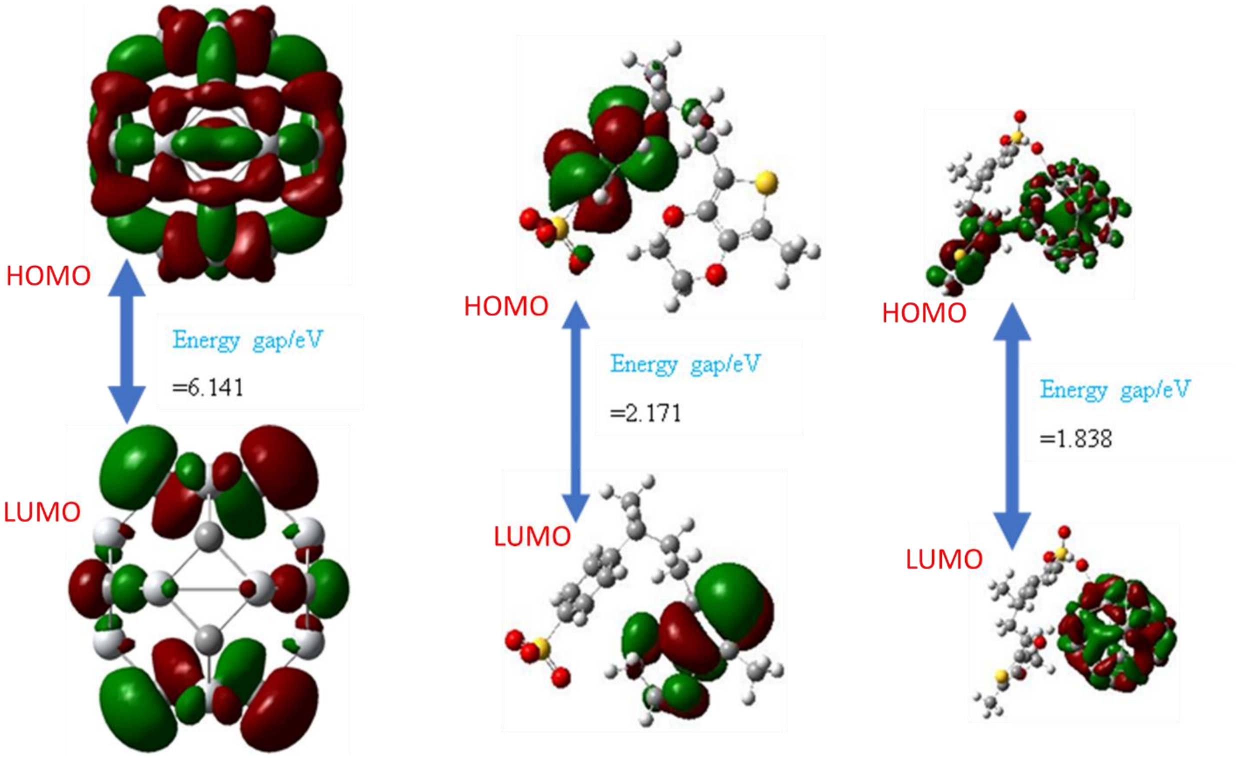

Figure S3. Pictorial representation of HOMO-LUMO energies and densities.

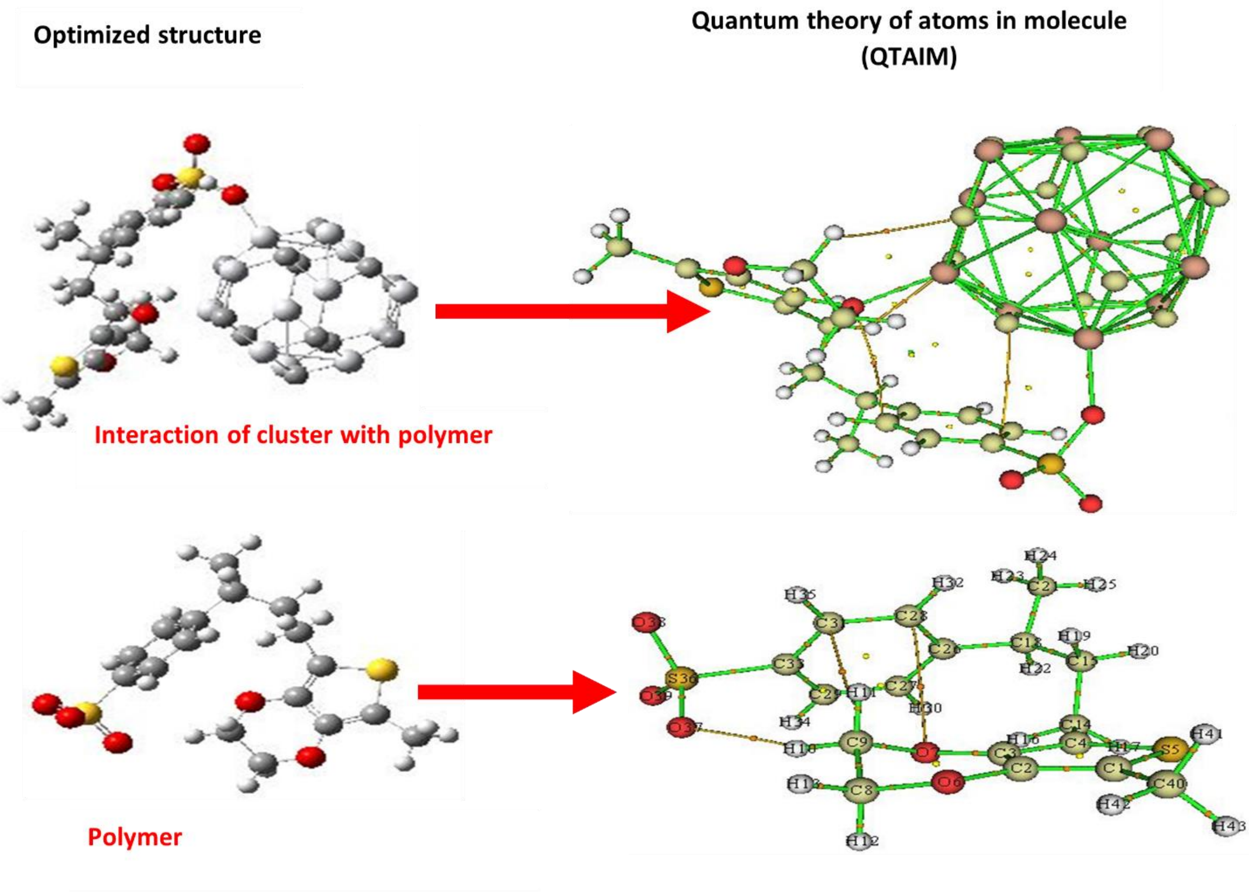

Figure S4. Pictorial representation of the Optimized structure and the QTAIM.

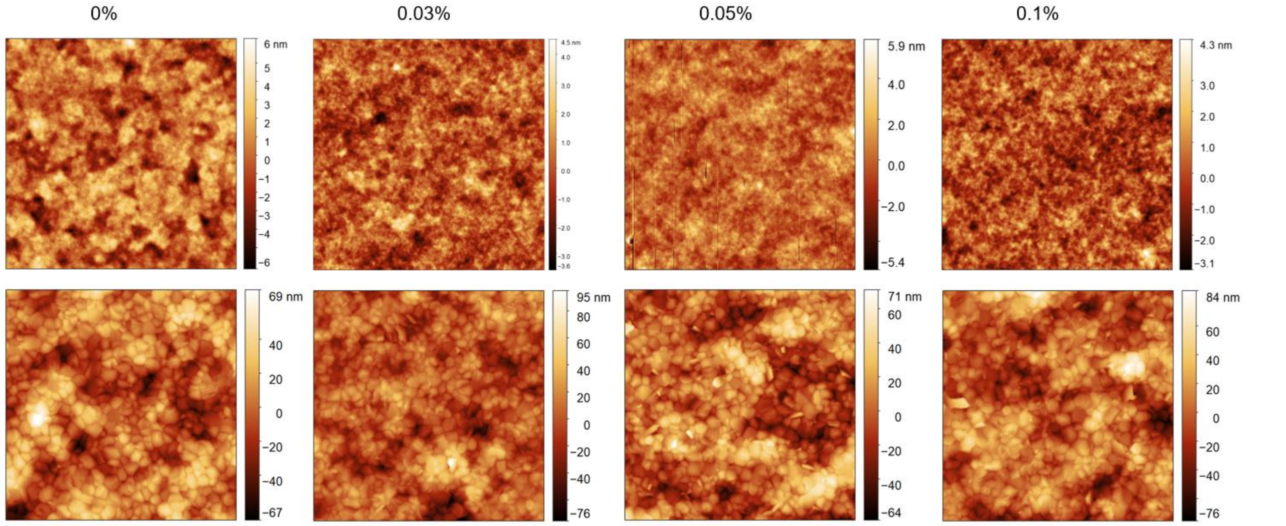

Figure S5. (top panel) AFM images of PEDOT:PSS films doped with various concentration of MXenes. (bottom panel) AFM images of perovskite films deposited on top of the same HTLs, respectively.

Table S1. Surface roughness of various HTL and perovskite films on HTLs.

Sample	Rms surface roughness (nm)
0 wt.% PEDOT:PSS	1.66
0.03 wt.% MXene – doped PEDOT:PSS	0.89
0.05 wt.% MXene – doped PEDOT:PSS	0.84
0.1 wt.% MXene – doped PEDOT:PSS	1.06
Perovskite on 0 wt.% PEDOT:PSS	19.22
Perovskite on 0.03 wt.% PEDOT:PSS	18.39
Perovskite on 0.05 wt.% PEDOT:PSS	19.84
Perovskite on 0.1 wt.% PEDOT:PSS	21.66

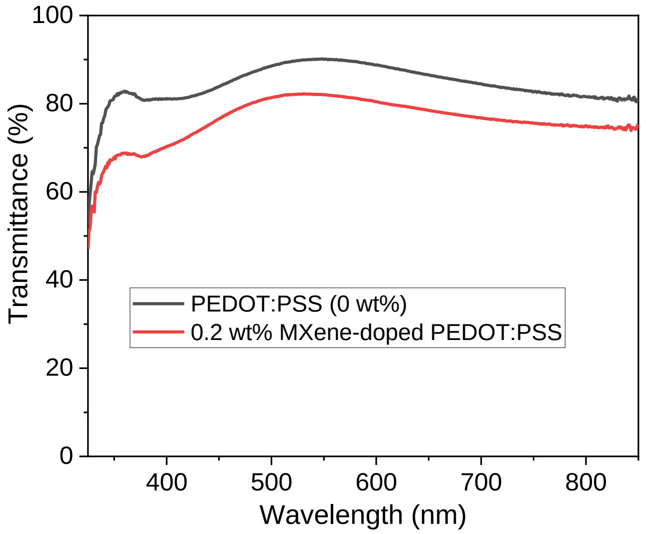

Figure S6. Transmittance spectrum of 0.2 wt.% doped MXene in PEDOT:PSS and a pristine PDOT:PSS layer on ITO.

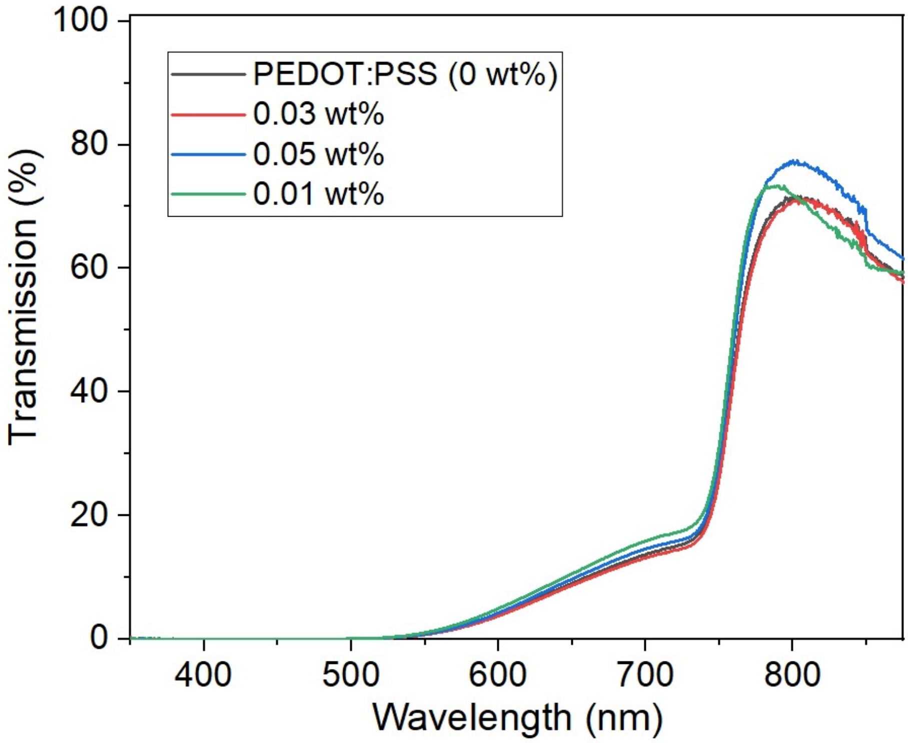

Figure S7. Transmission spectra of perovskite films on top of MXene-doped HTLs on ITO.

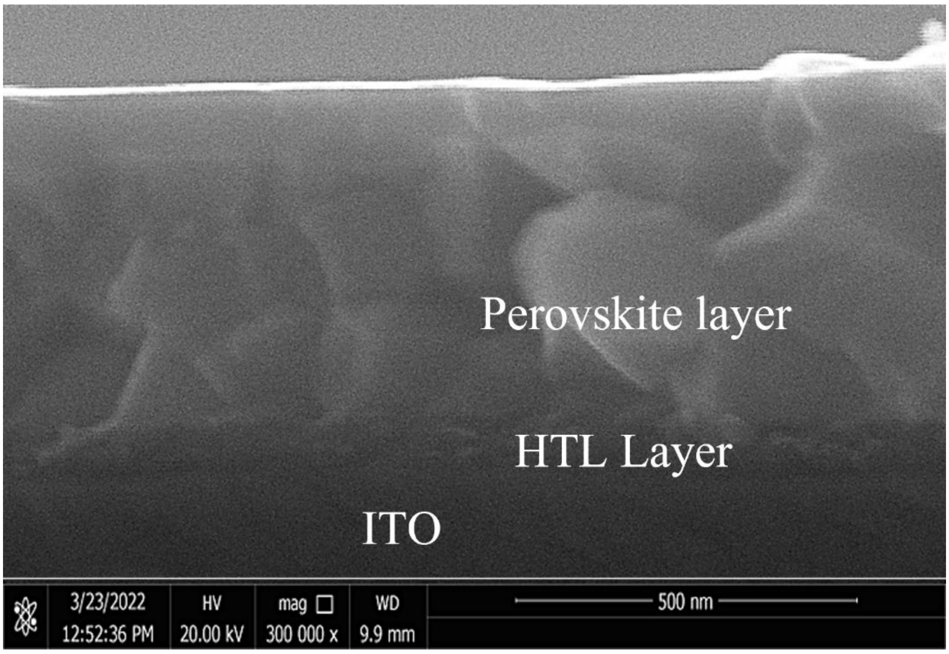

Figure S8. Cross-sectional SEM image of the device.

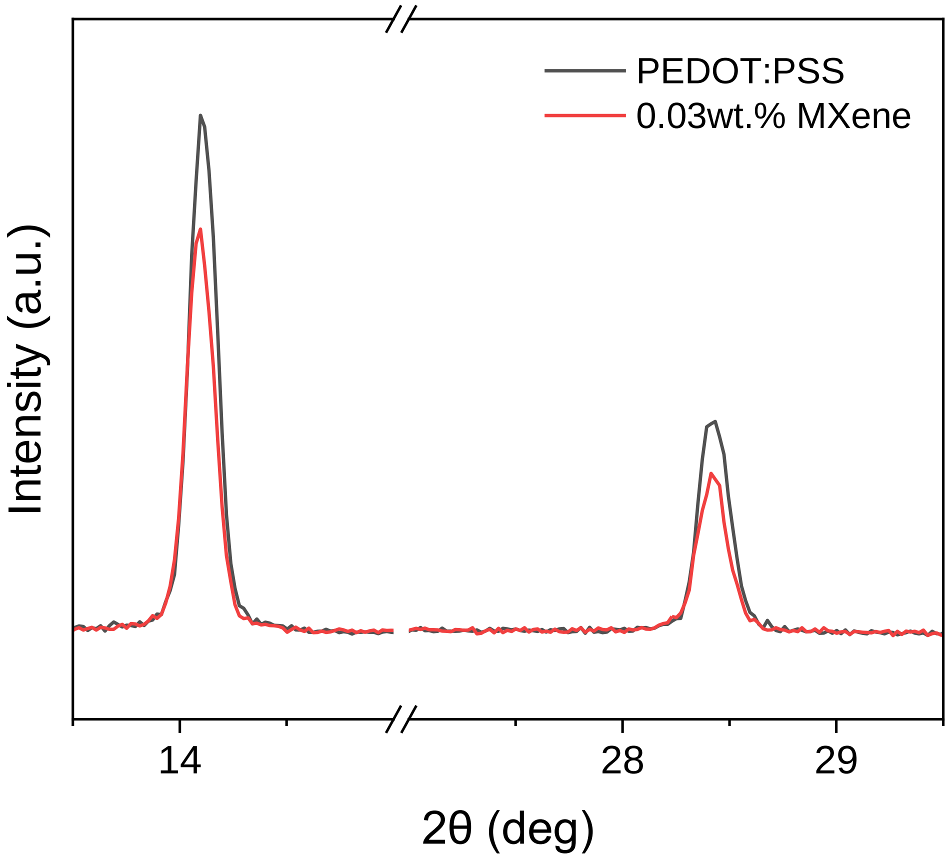

Figure S9: Comparison of the (110) and (220) XRD diffraction peaks.

Table S2. XRD peak analysis.

Sample	Peak label	Position (deg)	Intensity	Int. Ratio	Area	FWHM
PEDOT:PSS pristine	(110)	14.1	19.0	2.33	2.94	0.152
	(220)	28.4	8.1		1.38	0.167
0.03 wt.% MXene	(110)	14.1	15.0	2.39	2.32	0.155
	(220)	28.4	6.3		0.95	0.166
